# HbTGA1, a TGA Transcription Factor From *Hevea brasiliensis*, Regulates the Expression of Multiple Natural Rubber Biosynthesis Genes

**DOI:** 10.3389/fpls.2022.909098

**Published:** 2022-07-06

**Authors:** Dong Guo, Hui-Liang Li, Jia-Hong Zhu, Ying Wang, Shi-Qing Peng

**Affiliations:** ^1^Key Laboratory of Biology and Genetic Resources of Tropical Crops, Ministry of Agriculture, Institute of Tropical Bioscience and Biotechnology, Chinese Academy of Tropical Agricultural Sciences, Haikou, China; ^2^Key Laboratory for Biology and Genetic Resources of Tropical Crops of Hainan Province, Hainan Institute for Tropical Agricultural Resources, Haikou, China

**Keywords:** *Hevea brasiliensis*, TGA transcription factor, biosynthesis of natural rubber, gene, promoter

## Abstract

The TGA transcription factors are known to modulate the biosynthesis of secondary metabolites in plants. However, their regulatory function in natural rubber (NR) biosynthesis was not revealed in the rubber tree (*Hevea brasiliensis*). Here, 14 genes encoding TGA transcription factors (name *HbTGA1*-*HbTGA14*) were identified in the rubber tree. *HbTGAs* were differentially expressed in different tissues. *HbTGA1* was expressed at its highest level in latex. We found specific *in vitro* and *in vivo* binding of the HbTGA1 protein with promoters of multiple NR biosynthesis genes (*HbHMGS2*, *HbHMGR2*, *HbCPT6*, *HbCPT8*, and *HbSRPP2*). The activation of the promoters of *HbHMGS2* and *HbCPT6* was significantly suppressed by HbTGA1, while the activities of promoters of *HbHMGR2*, *HbCPT8*, and *HbSRPP2* were increased by HbTGA1. The promoter activities of *HbHMGS2*, *HbHMGR2*, *HbCPT6*, *HbCPT8*, and *HbSRPP2* were significantly increased by HbTGA1 under jasmonate stress, while the promoter activities of *HbHMGS2*, *HbHMGR2*, *HbCPT6*, *HbCPT8*, and *HbSRPP2* were also significantly increased by HbTGA1 under salicylic acid stress. The present study provides insights into the role of TGA transcription factors in regulating the expression of NR biosynthesis genes from *H. brasiliensis*.

## Introduction

Rubber tree (*Hevea brasiliensis* Muell. Arg) is an economically essential tropical tree and provides a sole commercial source of natural rubber (NR). NR, cis-1,4-polyisoprene, is an important industrial raw material because of its unique physical properties (Backhaus, 1985; van Beilen and Poirier, 2007). NR is an end-product of a side branch of the plant isoprenoid synthesis pathway (Yamashita and Takahashi, 2020). NR is produced in laticifer cells through a sequential condensation of isopentenyl diphosphates (IPPs) in a cis-1,4 configuration (Cornish, 2001; Chow et al., 2007; Salehi et al., 2021). As a consequence genes involved in IPP synthesis to cis-1,4-polyisoprene are named as NR biosynthesis genes (Tang et al., 2016). The biochemical pathways involving NR biosynthesis are now fully understood (Yamashita and Takahashi, 2020; Salehi et al., 2021), the regulatory mechanism of NR biosynthesis genes has not yet been fully investigated in the rubber tree.

The TGA transcription factors (TFs) are members of the subfamily of basic leucine zipper (bZIP) TFs (Li et al., 2019a; Wang et al., 2019). TGA proteins have the conserved bZIP domain-containing N-x_7_-R/K-x_9_-L-x_6_-L-x_6_-L motif, and Yx_2_RL[RQ]ALSS[LS]W motif, which is the unique motif of TGA TFs (Gatz, 2013). TGA TFs regulate gene expression by specific binding of TGACG-motif on gene promoter (Jakoby et al., 2002) and play crucial roles in secondary metabolites biosynthesis (Lv et al., 2019; Han et al., 2020; Huo et al., 2021), plant development (Wang et al., 2019), defense against pathogens (Kesarwani et al., 2007; Shearer et al., 2012), and responses to stress (Gatz, 2013; Li et al., 2019b). Given the potential importance of TGA TFs in plant secondary metabolites biosynthesis, we carried out the analysis of the TGA TFs family in the rubber tree and investigated the roles of TGA TFs in NR biosynthesis.

## Materials and Methods

### Identification of Rubber Tree TGA TFs

Ten Arabidopsis TGA protein sequences were downloaded from the NCBI database. These protein sequences were used as query sequences against the local rubber tree genome database (Li et al., 2017) through the BLASTp search (*E*-value 1.0 × e^−5^). Then, candidate rubber tree TGAs were further confirmed for conserved domains in the Pfam database (El-Gebali et al., 2018). The final confirmed genes were named *HbTGA1-HbTGA14*. The molecular weight and isoelectric point of HbTGAs were predicted by the ExPASy server (Gasteiger et al., 2003).

### Chromosomal Distribution of *HbTGAs*

The start and end points of *HbTGAs* were calculated from the rubber tree annotation GFF3 files, the chromosomal distribution of *HbTGAs* was displayed using MapChart v2.3.

### Analysis of Gene Structure and Construction of a Phylogenetic Tree

The exon and intron of *HbTGAs* were investigated using a web server GSDS (Hu et al., 2015). Multiple sequence alignments for HbTGA and other TGA proteins sequences from Arabidopsis, cassava, and *Ricinus communis* derived from the NCBI database were carried out using Clustal.^1^ The phylogenetic tree was constructed based on the HbTGA and other TGA protein sequences from Arabidopsis, cassava, and *R. communis* using MEGA7.0 software with Neighbor-Joining methods followed by 1,000 bootstrap replicates (Kumar et al., 2016).

### Expression Analysis of *HbTGAs*

The transcriptome data of different tissues, including latex (SRX1554800), bark (SRX1554797), leaf (SRX1554799), root (SRX1554786), female flower (SRX1554813), seed (SRX1554817), and male flower (SRX1554814), were obtained from the NCBI database. The expression levels of *HbTGAs* were calculated as fragments per kilobase of exon per million fragments mapped (FPKM), and heat-map was drawn based on the log_2_-transformed FPKM values using MeV 4.9.0 software.

### Prediction of TGA TF Binding Sites on the Promoters of NR Biosynthesis Genes

The 3.0 kb upstream sequences of the start codon of NR biosynthesis genes were obtained as the promoter region from the rubber tree local genome database. TGA TF binding sites on the promoters of NR biosynthesis genes were calculated using PlantCARE (Rombauts et al., 1999).

### Extraction of Rubber Tree DNA and RNA

DNA was isolated from the leaves according to method of Allen et al. (2006). RNA was extracted from latex as described previously (Tang et al., 2007).

### Subcellular Localization of HbTGA1

The coding sequence of *HbTGA1* was amplified with PCR primers (see Supplementary Table S1) from latex cDNA, and cloned into the *Bgl*II*/Nco*I restriction site of pCAMBIA1302, resulting in a HbTGA1 fused with green fluorescent protein (GFP) expression vector, which was further introduced into onion epidermal by *Agrobacterium*-mediated transformation. The transformed onion epidermal were incubated in darkness for 48 h at 25°C and then visualized under fluorescent microscopy.

### Yeast One-Hybrid

The promoter fragments of NR biosynthesis genes harboring TGACG-motif were obtained through PCR amplification with primers (see Supplementary Table S1), respectively. The bait vector was constructed by cloning the promoters of NR biosynthesis genes into the site of *Sac*I/*Spe*I of pHIS2.1 vector (Clontech). The coding sequence of *HbTGA1* was inserted into the pGADT7 vector at the site of *Eco*RI/*Bam*HI, generating prey vectors. Then, yeast Y187 strain was introduced with bait and prey vectors, respectively. The introduced yeast was cultured on SD/−Trp-Leu medium and SD/−Leu/−Trp/-His medium adding corresponding concentrations of 3-AT obtained by screening experiment for 3 days at 30°C.

### Electrophoretic Mobility Shift Assay

To determine the binding of the HbTGA1 protein with promoters of NR biosynthesis genes *in vitro*, electrophoretic mobility shift assay (EMSA) was performed. Firstly, the coding sequence of *HbTGA1* was cloned into the pET28a vector at the site of *Bam*HI/*Eco*RI, resulting in an HbTGA1-His-tagged protein expression vector. The expression vector was introduced into *Escherichia coli* BL21 (DE3). HbTGA1-His-tagged protein was purified using a HiTrap affinity column (GE Healthcare) in accordance with the instruction manual. Secondly, total 205 bp-long DNA fragments of the NR biosynthesis genes promoter (except HbHMGS2) were PCR-amplified, which containing the predicted TGACG-motifs with 100 bp upstream and 100 bp downstream. There were two TGACG-motifs between in 217 bp, total 417 bp-long DNA fragments of HbHMGS2 promoter was PCR-amplified, from 100 bp upstream of the first TGACG-motif to 100 bp downstream of the second TGACG-motif. The 205 bp-long DNA fragments which no TGACG-motif was PCR-amplified as negative control. The PCR products were purified using purification kit (Foregene, DE-03011). The primers used for EMSA are listed in Supplementary Table S1. Finally, EMSA was implemented according to the protocol of the EMSA kit (Invitrogen, E33075). In brief, the purified HbTGA1 was incubated with double-stranded promoter nucleotides for 30 min at room temperature. The DNA/protein complex samples were subjected to 12% polyacrylamide gel electrophoresis at 120 V for 30 min. Then, gel was examined using SYBR Green EMSA stain.

### Activation of the Promoters of NR Biosynthesis Genes by HbTGA1

To identify if HbTGA1 activates the promoters of NR biosynthesis genes in plant, a dual-luciferase assay system was employed for this purpose. In brief, *HbTGA1* was amplified and cloned into a pGreenII62 SK vector, generating an effector vector. The promoter of NR biosynthesis genes was amplified and cloned into a pGreenII0800-luciferase (LUC) vector, as a reporter vector. Then *Agrobacterium tumefaciens* GV3101 was introduced with effector or reporter vector. The *A. tumefaciens* containing the reporter and effector were mixed in the ratio of 1:2 (v:v) and then infiltrated into the tobacco following previously described method (Hellens et al., 2005). The infiltrated leaves were collected to measure LUC activities until 72 h after infiltration. For methyl jasmonate (MeJA) and salicylic acid (SA) treatment, tobacco leaves infiltrated with *A. tumefaciens* were sprayed with 100 μM MeJA, 100 μM SA, or water as control at 48 h after infiltration. The infiltrated leaves were collected and extracted protein for LUC activities assay at 24 h after treatment. The fluorescent values of LUC and the reference Renilla (REN) were measured in accordance with the instruction manual of the Dual-Luciferase Reporter Assay System (Promega, E1910). Experiments were performed in three biological replicates per treatment. Statistical analyses were carried out by one-way ANOVA (SAS6.11). Statistical significance was defined as *p* < 0.05.

## Results

### Identification and Characterization of HbTGAs

**Figure 1:**
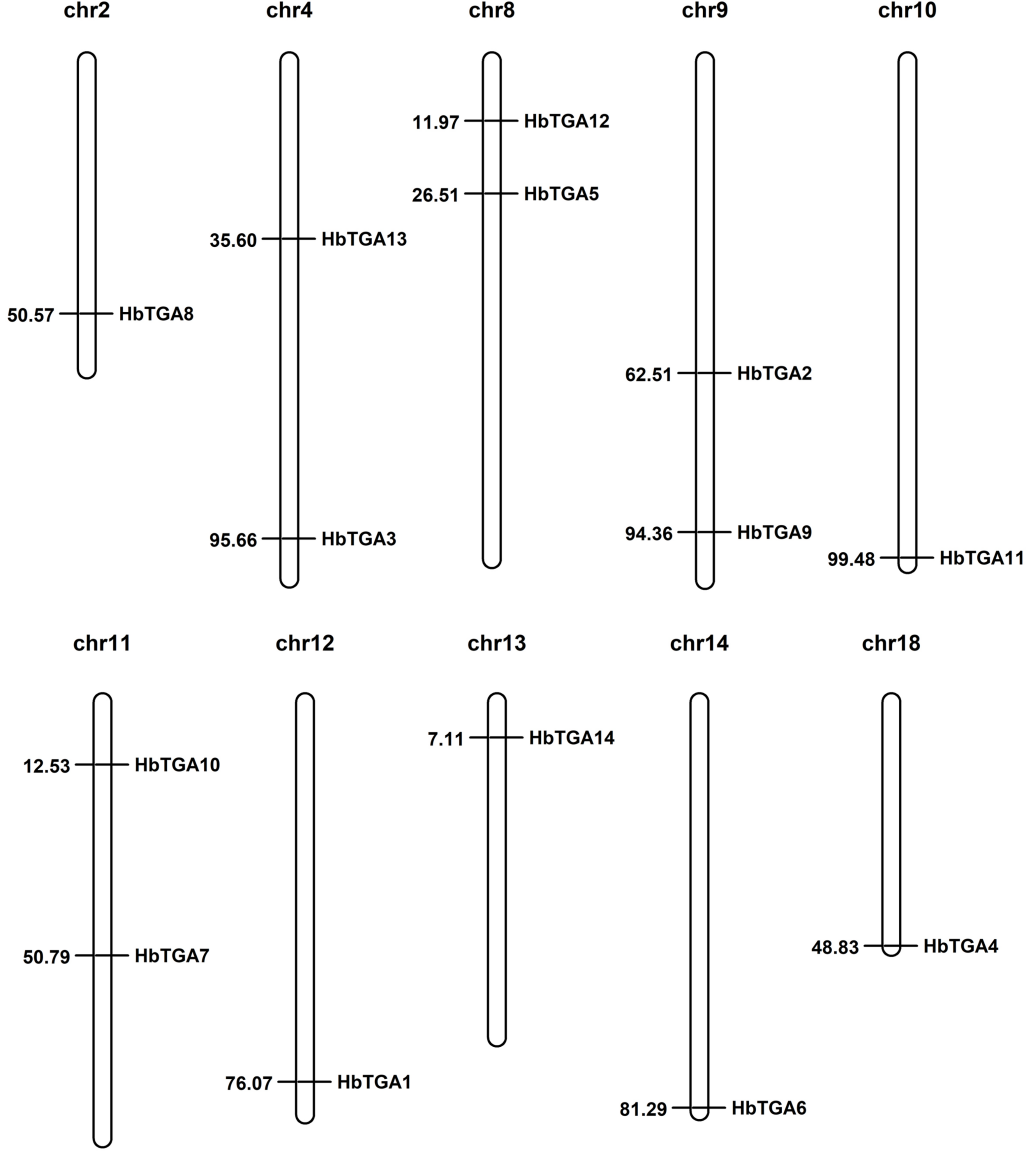
Chromosome location of *HbTGAs* in *Hevea brasiliensis*. 14 *HbTGAs* were localized onto 10 of 18 chromosomes. Map positions (in Mbp) are shown on the left.

A total of 14 putative genes encoding TGATFs were identified in the rubber tree genome. These genes were named as *HbTGA1*-*HbTGA14*. These predicted *HbTGAs* were distributed across 10 of 18 chromosomes (Figure 1). HbTGA proteins span 361 (HbTGA4) to 536 (HbTGA13) amino acids in length, with molecular weights spanning 40.62 kDa (HbTGA4) to 59.79 kDa (HbTGA13), and exhibit isoelectric points that range from 5.55 (HbTGA3) to 8.38 (HbTGA1; Table 1).

**Table 1:** Molecular characteristics of HbTGAs.

Gene ID	Name	Length of ORF	Number of aa	MW (kDa)	pI	Chromosome
LOC110671048	*HbTGA1*	1,122	373	41.96	8.38	12
LOC110661546	*HbTGA2*	1,404	467	51.49	7.71	9
LOC110666851	*HbTGA3*	1,398	465	51.4	5.55	4
LOC110647267	*HbTGA4*	1,086	361	40.62	8.29	18
LOC110635020	*HbTGA5*	1,407	468	51.64	7.25	8
LOC110661460	*HbTGA6*	1,368	455	50.23	6.5	14
LOC110641863	*HbTGA7*	1,353	450	49.72	6.36	11
LOC110651099	*HbTGA8*	1,131	376	42.27	8.37	2
LOC110648579	*HbTGA9*	1,566	521	58.57	6.73	9
LOC110637597	*HbTGA10*	1,479	492	54.48	7.24	11
LOC110653843	*HbTGA11*	1,386	461	50.97	6.69	10
LOC110663529	*HbTGA12*	1,560	519	58.13	6.82	8
LOC110659950	*HbTGA13*	1,611	536	59.79	6.55	4
LOC110640901	*HbTGA14*	1,122	373	41.83	7.53	13

**Figure 2:**
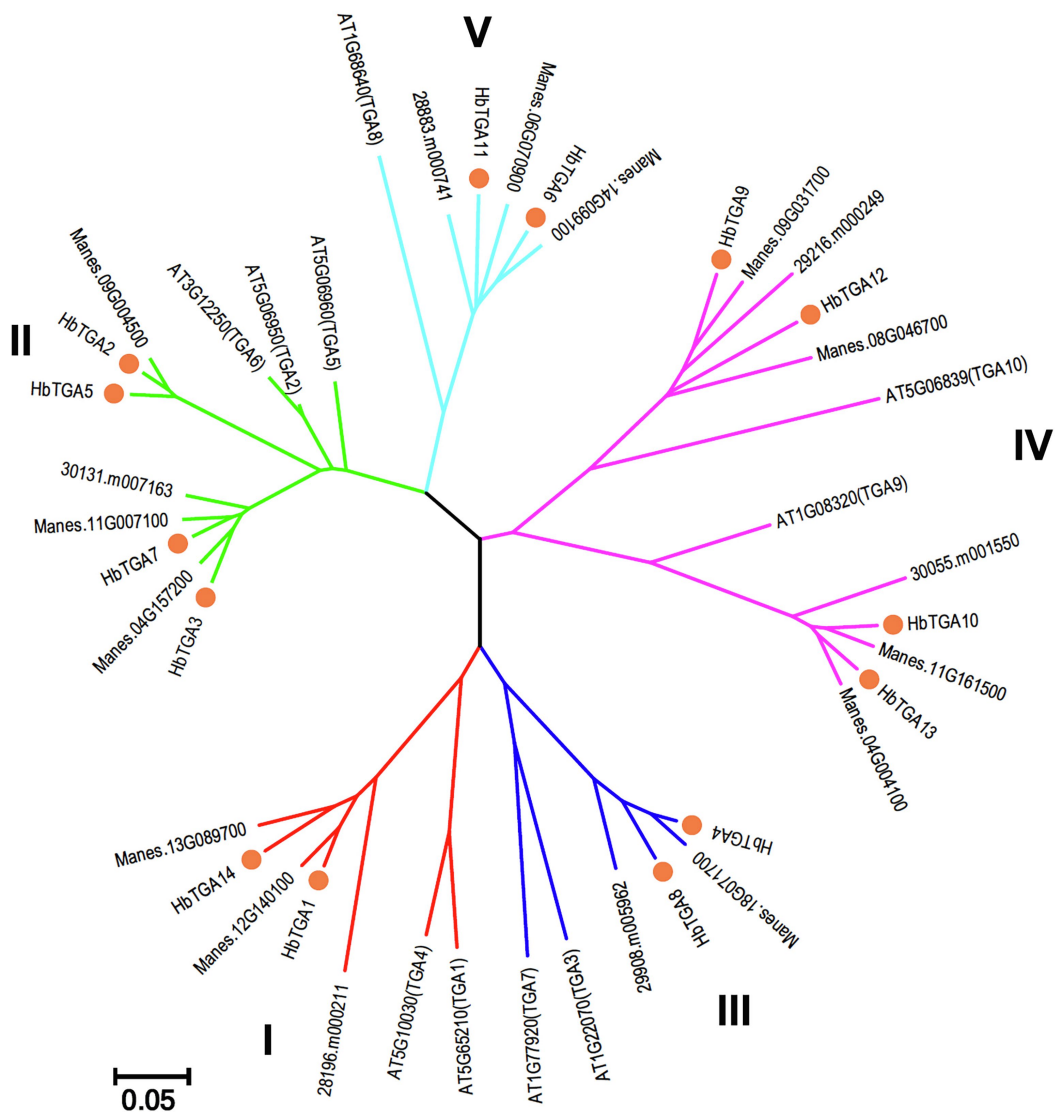
Phylogenetic analysis of TGA proteins from Arabidopsis, cassava, and *Ricinus communis* with Neighbor-Joining method using 1,000 bootstrap replicates. Each clade is demonstrated by different color.

To explore the evolutionary relationships between HbTGAs and TGAs from other plants, a phylogenetic tree was constructed by comparing HbTGAs with TGA proteins from Arabidopsis, cassava, and *R. communis*. As shown in Figure 2, 42 TGA proteins were separated into five clades. Clades II and IV both contain four HbTGAs, while clade I, III, and V have two members of the HbTGAs family.

**Figure 3:**
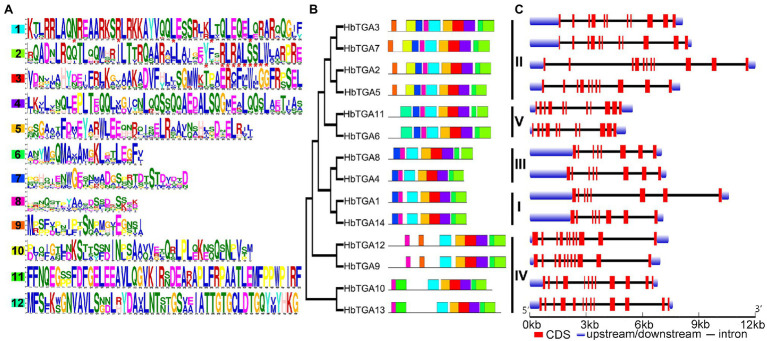
Conserved motifs, phylogenetic tree, and gene structure in *HbTGAs*. **(A)** Conserved motifs of the TGA proteins. Each motif is represented by a number in colored box. **(B)** Phylogenetic relationships. **(C)** Gene structure in *HbTGAs*.

The conserved motifs of all the 14 HbTGAs were found (Figure 3A) using MEME online tool using “searching for 12 motifs.” As shown in Figure 3B, all 14 HbTGAs contained motifs 1, 2, 3, 4, 5, 6, and 8. Motif 1(N-x7-R/K-x9-L-x6-L-x6-L) is a typical bZIP domain in b-zip TFs and motif 2(Yx2RL[R/Q]ALSS [L/S]W) is a unique bZIP-D box in TGA TFs. Except for HbTGA9, 10, 12, and 13, other TGAs contain motif 7. Except for HbTGA2, 3, 5, and 7, 9, 12, other TGAs do not contain motif 9. HbTGA2, 3, 5, and 7 have motif 10. HbTGA10 and HbTGA13 have motif 11. HbTGA6 and HbTGA11 have motif 12.

The gene structures of 14 *HbTGAs* were also examined. *HbTGAs* were interrupted by more than six introns (Figure 3C). *HbTGAs* in Clades II, IV, and V contain more than 10 introns, while *HbTGAs* in Clades I and II have seven introns. The same group of *HbTGAs* has a similar gene structure and the same conserved domain, which may reflect their similar functions.

### Expression Patterns of *HbTGAs*

**Figure 4:**
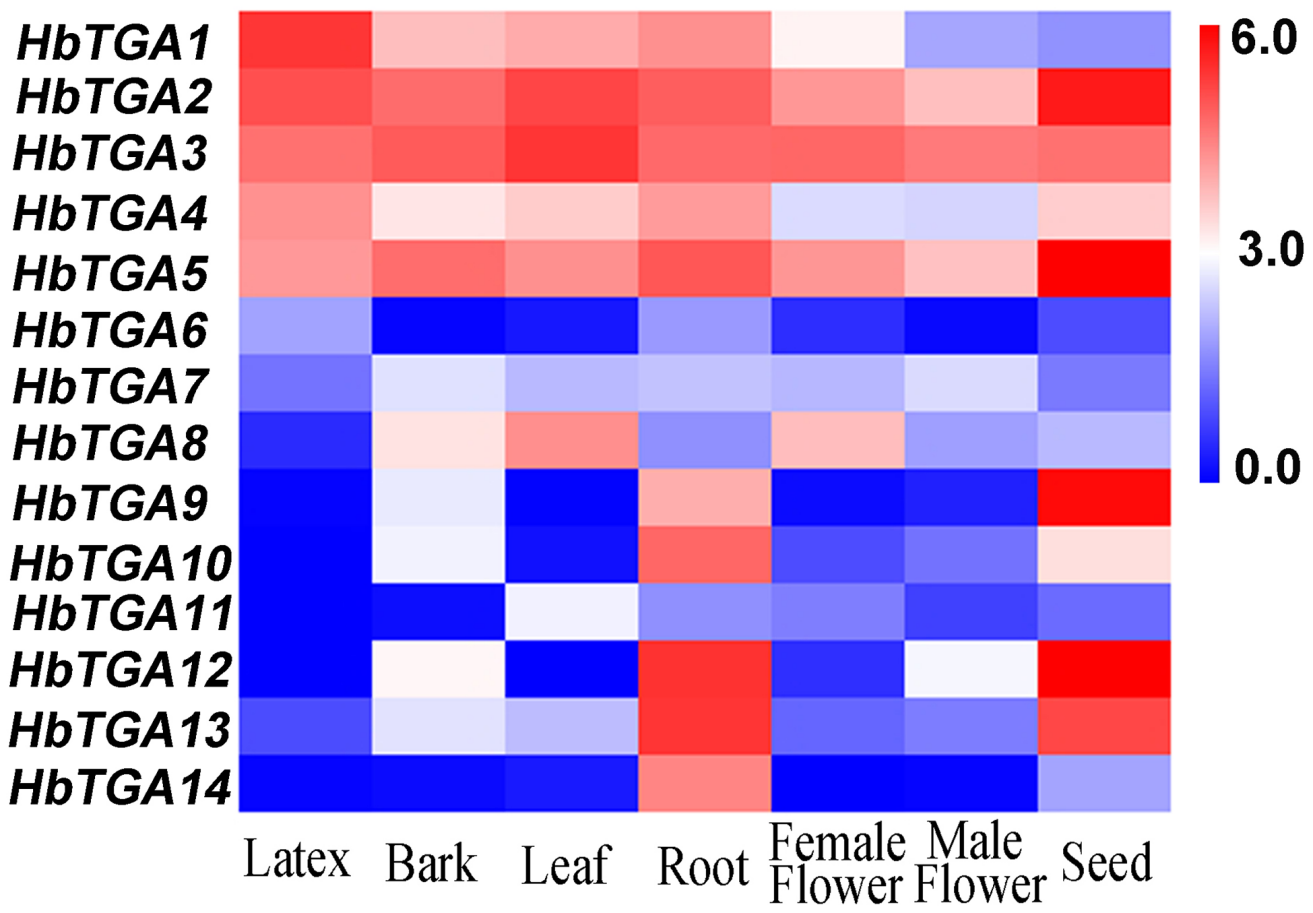
Expression patterns of HbTGAs in different tissues. The heat-map was created using log2-transformed fragments per kilobase of exon per million fragments mapped (FPKM) values from the transcriptome data of different tissues, including latex, bark, leaf, root, female flower, and male flower seed.

The expression patterns of *HbTGAs* were analyzed using the transcriptome data of different rubber tree tissues (Figure 4). All the 14 *HbTGAs* were expressed in the evaluated tissues, but there were differences in expression patterns. For example, the expression of *HbTGA1* showed the highest level in latex. *HbTGA2* and *HbTGA3* had higher expression levels in all evaluated tissues, while *HbTGA6*, *HbTGA7*, and *HbTGA11* had lower expression levels in all evaluated tissues. *HbTGA12* and *HbTGA13* were expressed most strongly in the root, *HbTGA2*, *HbTGA5*, *HbTGA9*, and *HbTGA12* were expressed at their highest levels in the seed. In addition, *HbTGA10*, *11*, *12* were not expressed in latex, *HbTGA12* was not expressed in leaf, while *HbTGA12* was not expressed in the female flower.

### Prediction of the Binding Site of TGA TFs on the Promoters of NR Biosynthesis Genes

**Figure 5:**
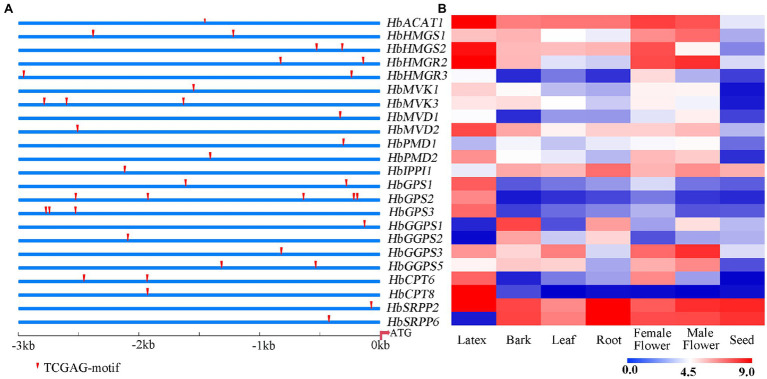
Prediction of binding site of TGA TFs on the promoters of NR biosynthesis genes **(A)** and expression patterns of NR biosynthesis genes in different tissues **(B)**. The heat-map was created using log2-transformed FPKM values from the transcriptome data of different tissues, including latex, bark, leaf, root, female flower, male flower seed.

The 3 kb upstream fragment of the start codon (ATG) of NR biosynthesis-related gene was isolated as the promoter region. The putative TGA TFs binding site (TGACG-motif) was predicted on the promoters of NR biosynthesis genes. As shown in Figure 5A, the promoters of these natural rubber synthesis genes contain 1–3 TGA TFs recognition sites. The expression of NR biosynthesis genes was analyzed using the transcriptome data of seven rubber tree tissues and organs (Figure 5B). According to the expression abundance of these genes in latex, *HbHMGS2*, *HbHMGR2*, *HbCPT6*, *HbCPT8*, and *HbSRPP2* which were expressed at a higher level in latex were selected to further verify whether TGA TFs interact with promoters of NR biosynthesis genes.

### HbTGA1 Is a Nuclear Protein and Interacts With Promoters of NR Biosynthesis Genes

**Figure 6:**
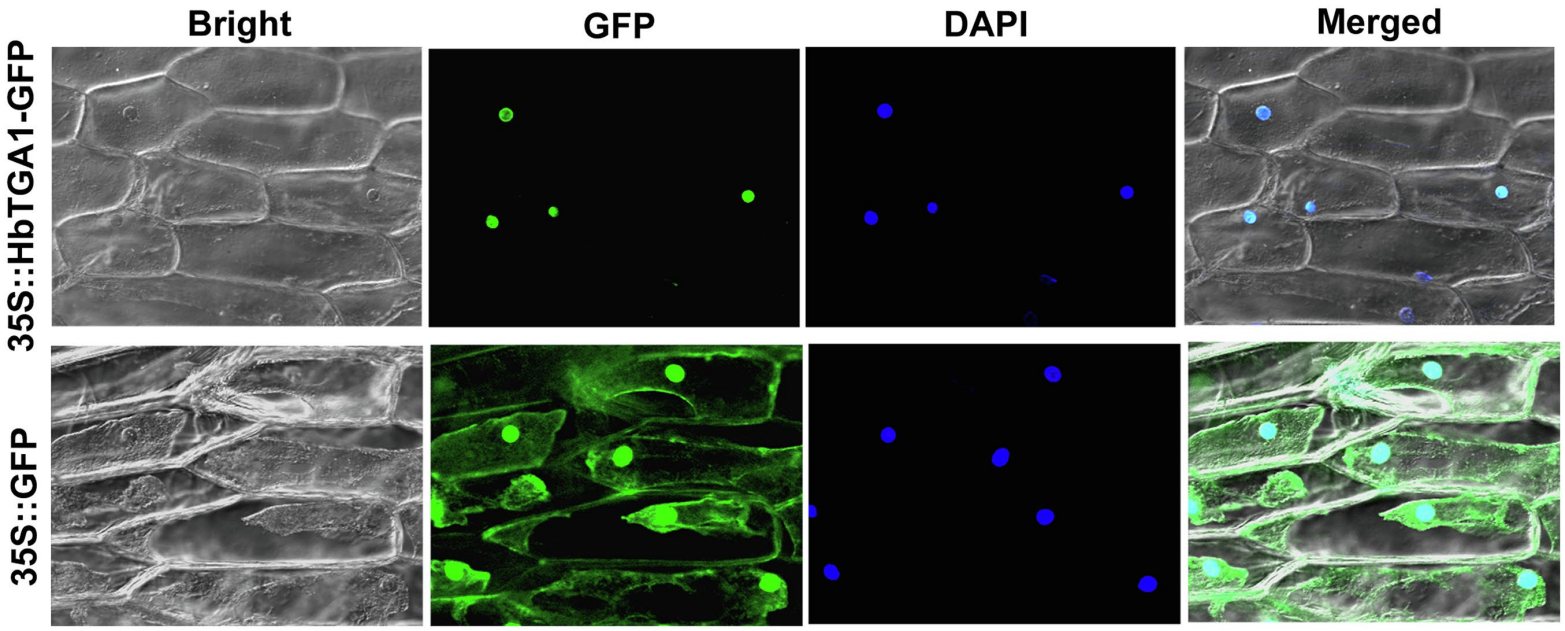
Subcellular localization of HbTGA1. Transient expression of 35S::HbTGA1-GFP (upper layer) and 35S::GFP (under layer, control vector) in onion epidermal cells, showing the nuclear localization of HbTGA1.

**Figure 7:**
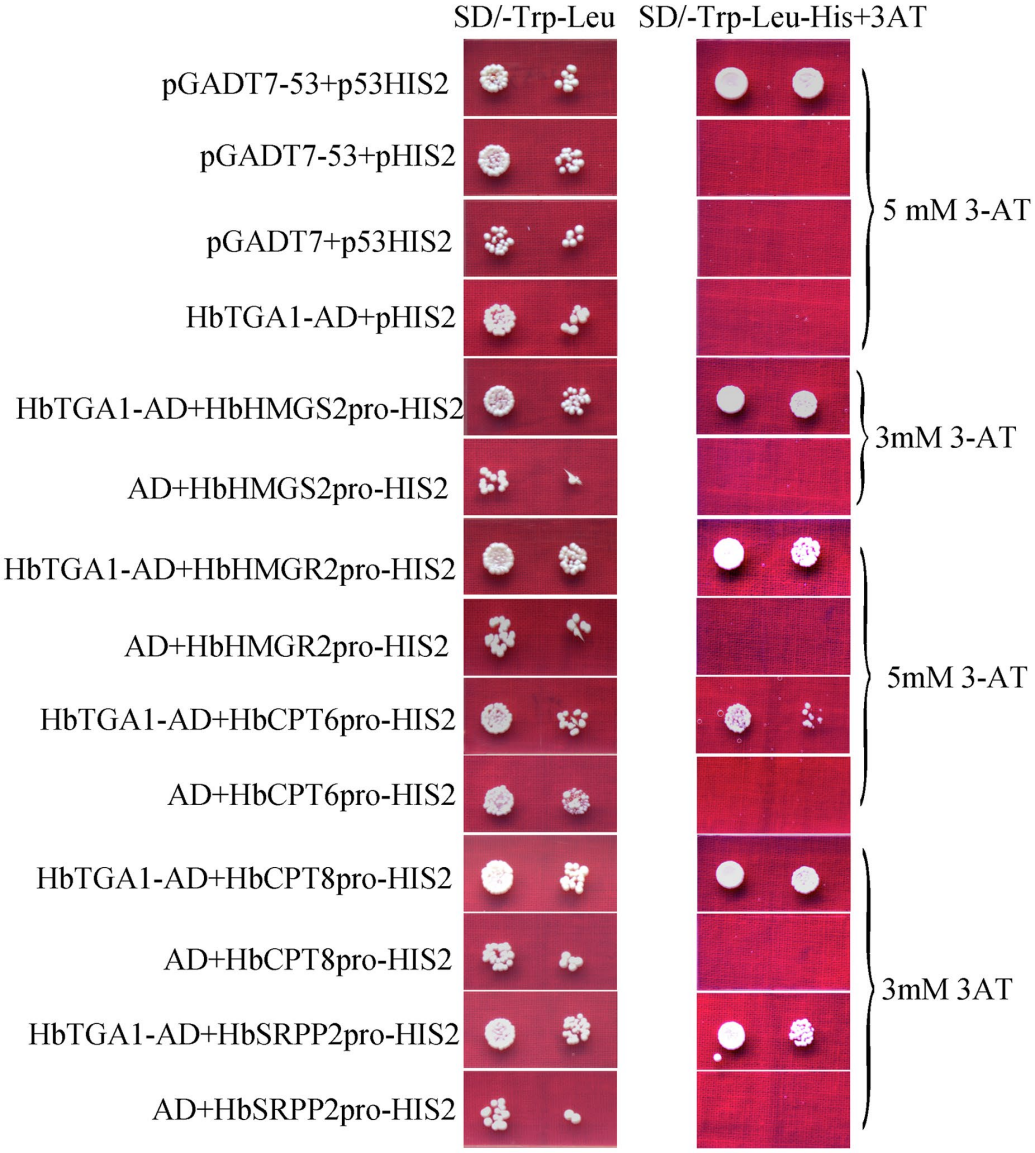
HbTGA1 binding with the promoters of NR biosynthesis genes in yeast. Yeast harboring bait and prey vectors were grown in SD/−Trp-Leu medium and SD/−Leu/−Trp/-His selective medium adding corresponding concentrations of 3-AT obtained by screening experiment for 3 days at 30°C.

**Figure 8:**
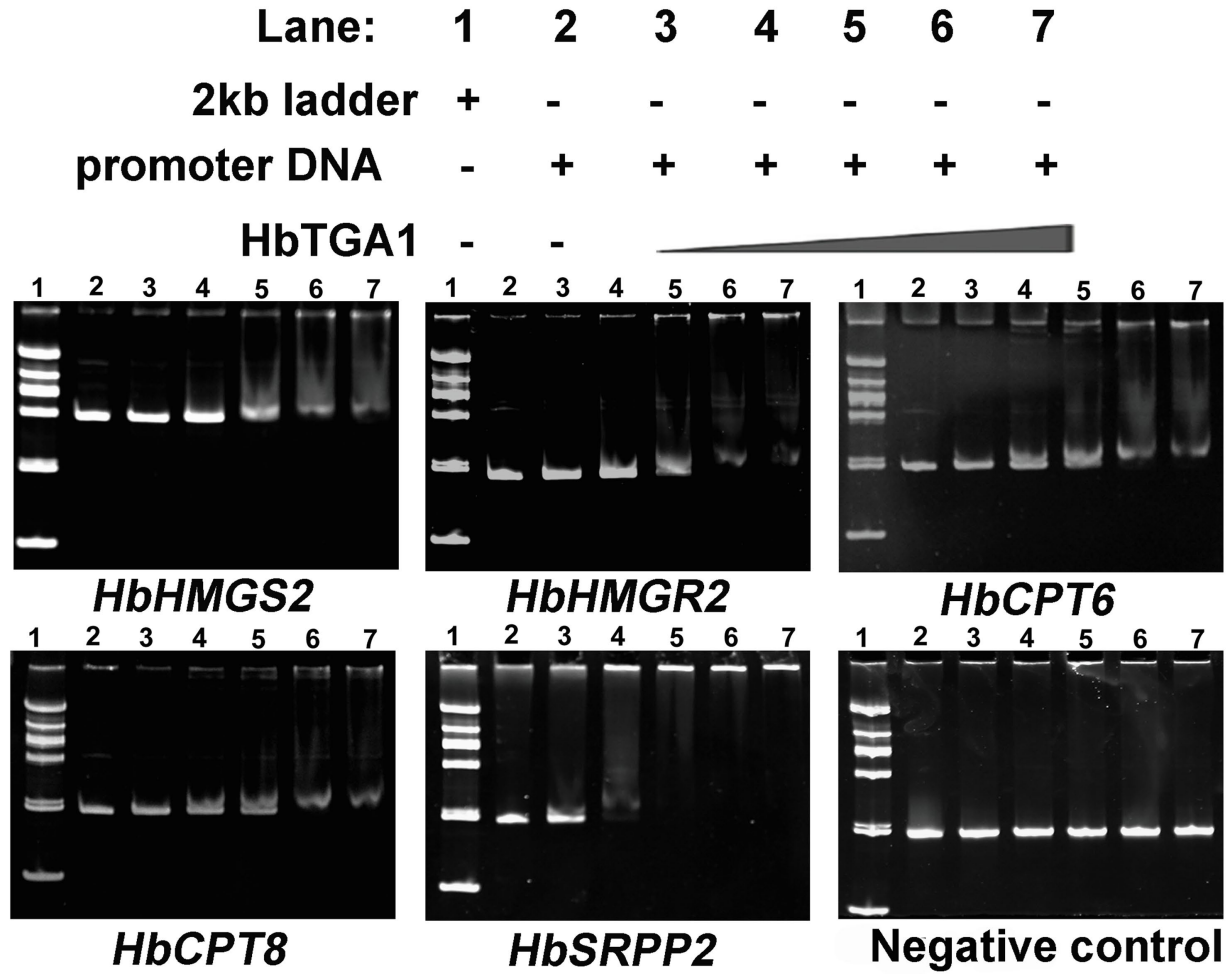
HbTGA1 binding with the promoters of NR biosynthesis genes was analyzed *via* electrophoretic mobility shift assay (EMSA). Lane 1: DNA 2,000 markers. Lane 2: 50 ng, the promoter of NR biosynthesis genes only. Lanes 3–7: 50 ng, the promoter of NR biosynthesis genes with increasing amounts of HbTGA1 protein (100, 200,300, 400, and 500 ng).

The subcellular localization of HbTGA1 was analyzed in transiently transformed onion epidermal cells. As shown in Figure 6, HbTGA1 was located in the nucleus. The binding specificity of these promoters of NR biosynthesis genes with HbTGA1 was determined by the yeast one-hybrid experiment (Figure 7). An *in vitro* EMSA assay further suggested HbTGA1 bound with promoters of *HbHMGS2*, *HbHMGR2*, *HbCPT6*, *HbCPT8*, and *HbSRPP2* (Figure 8). This finding indicates that HbTGA1 binds to the promoters of these NR biosynthesis genes and activates the transcription in yeast.

### HbTGA1 Regulated the Activation of Promoters of Multiple NR Biosynthesis Genes

**Figure 9:**
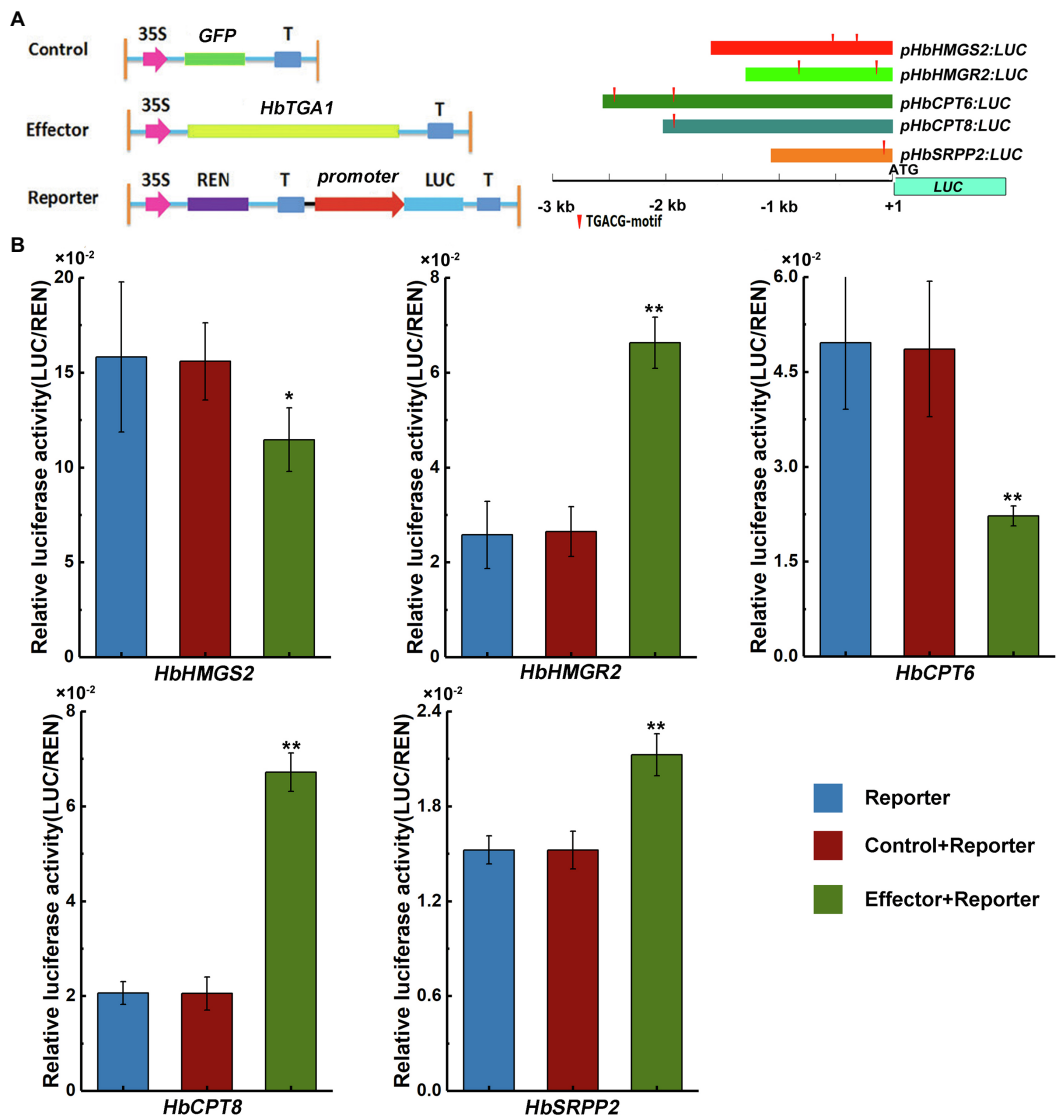
Effect of HbTGA1 on the activation of the promoter of NR biosynthesis genes. Schematic maps of e-effector and reporter **(A)**. The promoter activity was determined by a transient dual-luciferase (LUC) assay **(B)**. The relative LUC activities (LUC/REN) were normalized to the reference *Renilla* (REN) LUC. Error bars represent SD of three technical replicates. *p < 0.05, **p < 0.01.

Since NR is synthesized in laticifers, genes highly expressed in laticifers may participate in NR biosynthesis (Oh et al., 1999). The expression level of *HbTGA1* is the most highest among all *HbTGAs*. To investigate whether HbTGA1 involves in regulating NR biosynthesis-related genes, we transiently expressed *HbTGA1* along with a *LUC* controlled by the promoters of NR biosynthesis genes in tobacco. The schematic diagrams of the effectors and reporters used in dual-luciferase assay system are shown in Figure 9A. Compared to the control, the expression of *HbTGA1* enabled to change the level of the LUC activity controlled by the promoters of NR biosynthesis genes. As shown in Figure 9B, the activation of the promoter of *HbHMGS2*, and *HbCPT6* were significantly suppressed by HbTGA1, decreased by 27 and 55%, respectively; while the activation of promoters of *HbHMGR2*, *HbCPT8*, and *HbSRPP2*, increased to 2.5-, 3.3-, and 1.4-fold, respectively (Figure 9B). Taken together, HbTGA1 takes part in regulating the expression of multiple genes in the NR synthesis pathway.

**Figure 10:**
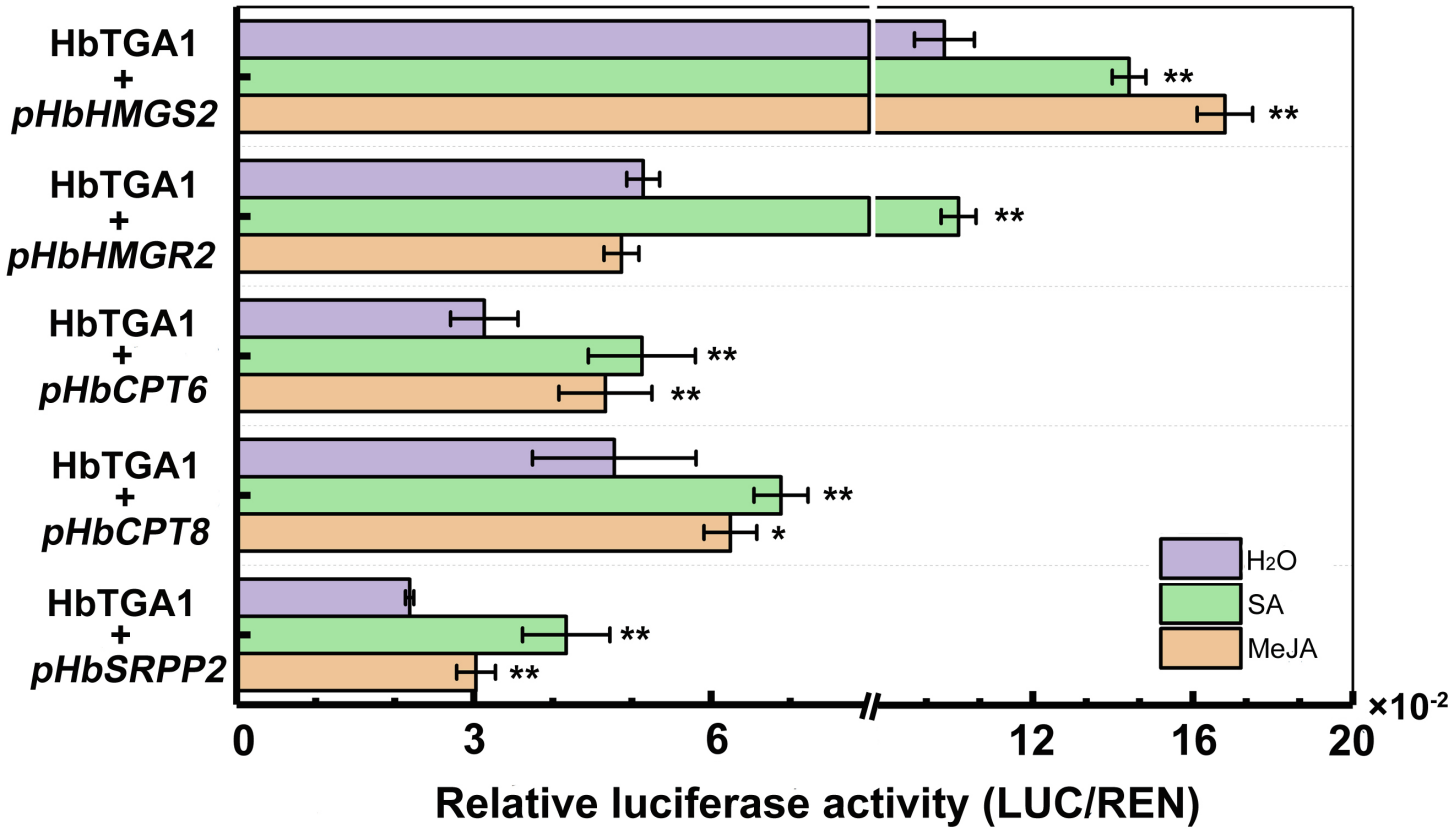
Activation of the promoter of NR biosynthesis genes by HbTGA1 under JA and SA. Error bars represent SD of three technical replicates. *p < 0.05, **p < 0.01.

Subsequently, we transiently expressed *HbTGA1* along with a *LUC* controlled by the promoters of NR biosynthesis genes in tobacco under MeJA and SA treatment conditions (Figure 10). Compared to the control, the promoter activities of *HbHMGS2* and *HbCPT6*, *HbCPT8*, and *HbSRPP2* were significantly increased by HbTGA1 under MeJA stress, increased to 1.5-, 1.5-, 1.3-, and 1.4-fold, respectively; the promoter activities of *HbHMGS2*, *HbHMGR2*, *HbCPT6*, *HbCPT8*, and *HbSRPP2* were also significantly increased by HbTGA1 under SA stress, increased to 1.3-, 2.0-, 1.6-, 1.4-, and 1.9-fold, respectively. The result revealed that HbTGA1 might participate in the MeJA- and SA-inducible NR biosynthesis in rubber tree.

## Discussion

A number of investigates have proved the involvement of TGA TFs in several biological processes (Shearer et al., 2012; Gatz, 2013; Canales et al., 2017; Sun et al., 2017; Lv et al., 2019; Wang et al., 2019; Han et al., 2020; Huo et al., 2021). However, the TGA TFs family and their role in the rubber tree were scarcely understood. Here, we identified 14 TGA TFs members in the rubber tree. The HbTGAs could be clustered together with TGAs from Arabidopsis, cassava, and *R. communis* in the same clade, suggesting that the evolution of TGA genes is conserved.

*Transcription factors regulate* gene expression and play key roles in *biological* processes. In rubber tree, a few TFs take part in regulation of the expression of NR biosynthesis genes. For example, three TFs, including HbWRKY1, HbWRKY14, and HbMADS4, downregulated the expression of *HbSRPP* (Wang et al., 2013; Li et al., 2016, 2020). HbCZF1 upregulates the expression of *hmg1* (Guo et al., 2015). HblMYB19, HblMYB44, and HbWRKY27 upregulated the expression of *HbFPS1* (Wang et al., 2017; Qu et al., 2020). HbRZFP1 downregulated *HRT2* expression (Guo et al., 2018). HbMYC2b activates *HbSRPP* expression (Guo et al., 2019). These data showed TFs play critical roles in NR biosynthesis. It was found that TGA TFs regulate plant secondary metabolites biosynthesis. In *Artemisia annua*, AaTGA6 regulates artemisinin content by directly binding to the promoter of *AaERF1*. In *Tripterygium wilfordii*, TwTGA1 binds with promoters of *PMT* and *MPO1* and activates their expressions, and modulates secondary metabolites biosynthesis (Han et al., 2020). In our study, Y1H and EMSA assays showed that HbTGA1 bound to the promoter of NR biosynthesis genes. The activation of promoters of multiple NR biosynthesis genes was regulated by HbTGA1. These results suggested HbTGA1 might modulate the expression of NR biosynthesis genes and participate in NR synthesis in rubber tree. The functions of HbTGA1 participating in NR synthesis in rubber tree needs to be explored in rubber tree in future. Additionally, TGA factors have been shown to interact with other TFs to modulate their activity (Gatz, 2013), the potential regulators of HbTGA1 also needs to further be investigated.

In plant TGA, TFs have key roles in secondary metabolites biosynthesis through SA and JA signaling pathways (Lv et al., 2019; Huo et al., 2021). For example, TwTGA1 was reported to increase the MeJA-inducible triptolide synthesis by upregulating the expression of *TwTPS27a/b* in *T. wilfordii* (Han et al., 2020; Huo et al., 2021). AaTGA6 was reported to modulate SA-inducible artemisinin synthesis in *A. annua* (Lv et al., 2019). In this study, the activation of promoters of multiple NR biosynthesis genes was significantly increased by HbTGA1 under SA and JA. In addition, SA could also induce a transient increase in NR yield in rubber tree (Tungngoen et al., 2011), suggesting SA signaling pathway might play role in regulating NR biosynthesis. However, there is still a lack of direct evidence to show SA signaling regulatory involvement in NR biosynthesis and the core module of SA signaling needs to further clarify in rubber tree. JA signaling has been reported to modulate NR biosynthesis in rubber tree (Deng et al., 2018). Being the target of SA signaling, HbTGA1 can connect the SA pathway with the JA pathway (Gatz, 2013). Thus, it will be of great interest to further study the regulatory mechanisms of HbTGA1 modulating MeJA- and SA-inducible NR biosynthesis. HbTGA1 might become a biotechnological tool in rubber tree breeding.

## Data Availability Statement

The original contributions presented in the study are included in the article/Supplementary Material; further inquiries can be directed to the corresponding author.

## Author Contributions

S-QP: conceptualization. DG, H-LL, J-HZ, and YW: investigation. DG and S-QP: writing—original draft and writing—review and editing. All authors contributed to the article and approved the submitted version.

## Funding

This study was supported by Hainan Provincial Natural Science Foundation of China (No. 322MS125), National Natural Science Foundation of China (No. 31970620), and Central Public-Interest Scientific Institution Basal Research Fund for Chinese Academy of Tropical Agricultural Sciences (No. 1630052022009).

## Conflict of Interest

The authors declare that the research was conducted in the absence of any commercial or financial relationships that could be construed as a potential conflict of interest.

## Publisher’s Note

All claims expressed in this article are solely those of the authors and do not necessarily represent those of their affiliated organizations, or those of the publisher, the editors and the reviewers. Any product that may be evaluated in this article, or claim that may be made by its manufacturer, is not guaranteed or endorsed by the publisher.

## Footnotes

^1^ http://www.clustal.org/
