# Supplementary material for: HbTGA1, a TGA Transcription Factor From Hevea brasiliensis, Regulates the Expression of Multiple Natural Rubber Biosynthesis Genes
Source: Front Plant Sci. 2022 Jul 6;13:909098. doi: 10.3389/fpls.2022.909098 (PMC9297914; doi:10.3389/fpls.2022.909098)
Supplement: Supplementary file 1 [file Table_1.docx]

**Table S1 List of primers used in this research**

| **Gene** | **Forward primer(5'-3')** | **Reverse primer (5'-3')** | **Restriction site** | **Vector** | **Usage** |
| --- | --- | --- | --- | --- | --- |
| *HbTGA1* | ATCCATGGTAATGAACTCTCCGTCTACACAGTTTG | ATAGATCTGCAGGCTCACGAGGACGAGTCACCC | *Nco*I/*Bgl*II | pCAMBIA1302 | Subcellular localization |
| *HbTGA1* | ATGGATCCATGAACTCTCCGTCTACACAGTTTG | ATGAATTCCTAGGCAGGCTCACGAGGACGAGTC | *Bam*HI/*Eco*RI | pET28a | protein expression |
| *HbTGA1* | ATGAATTCATGAACTCTCCGTCTACACAGTTTG | ATGGATCCCTAGGCAGGCTCACGAGGACGAGTC | *Eco*RI/*Bam*HI | pGADT7/pGreenII 62SK | Y1H/effector vector |
| *HbHMGS2* | GCGAGCTCTAACTAATTTTAATATAATAACTTA | GCACTAGTAAGAGAGACTGAGAAGGATGATTAT | *Sac*I/*Spe*I | pHIs2.1 | Y1H |
| *HbHMGR2* | GCGAGCTCATAATAATTTTTTTTAATGTTGGTG | GCACTAGTATACAAAACCCCAAATAAATTAATT | *Sac*I/*Spe*I | pHIs2.1 | Y1H |
| *HbCPT6* | GCGAGCTCGGAGTTTGGTATGCAATATTTAAGA | GCACTAGTTTATAAGTCTAATTTCTTTATTGTA | *Sac*I/*Spe*I | pHIs2.1 | Y1H |
| *HbCPT8* | GCGAGCTCTTTTGGAGTTATGCAATATTTAAGA | GCACTAGTAATTAAGTCTAATTTCTTTATTGTA | *Sac*I/*Spe*I | pHIs2.1 | Y1H |
| *HbSRPP2* | GCGAGCTCTGCAACTGGTTTTCACACGCCTTTT | GCACTAGTGATTGCGGAAATGGAAGATCAAAAA | *Sac*I/*Spe*I | pHIs2.1 | Y1H |
| *HbHMGS2* | GTCGACGCCACTCCTGGATTGAAATGGGTTGG | CTGCAGTTCTCTACGCCTCTCCAATTCCTCCT | *Sal*I/*Pst*I | pGreenII 0800 | reporter vector |
| *HbHMGR2* | GTCGACGAGTTACAGGCTGAGGGATGAAAAGA | CTGCAGGTAAAAATATGCCGGCGCAGGAGAGA | *Sal*I/*Pst*I | pGreenII 0800 | reporter vector |
| *HbCPT6* | GTCGACGGAGTTTGGTATGCAATATTTAAGAT | CTGCAGTTTACTTAAACCACTGACTTAACCTG | *Sal*I/*Pst*I | pGreenII 0800 | reporter vector |
| *HbCPT8* | GTCGACTTTTGGAGTTATGCAATATTTAAGAT | CTGCAGTTTCCTTAAACCACTGACTTAACCTG | *Sal*I/*Pst*I | pGreenII 0800 | reporter vector |
| *HbSRPP2* | GTCGACATAGGGATGGCAATGGGCAGGACATT | CTGCAGGATTGCGGAAATGGAAGATCAAAAAT | *Sal*I/*Pst*I | pGreenII 0800 | reporter vector |
